# Synthesis, detailed geometric analysis and bond-valence method evaluation of the strength of π-arene bonding of two isotypic cationic prehnitene tin(II) complexes: [{1,2,3,4-(CH_3_)_4_C_6_H_2_}_2_Sn_2_Cl_2_][*M*Cl_4_]_2_ (*M* = Al and Ga)

**DOI:** 10.1107/S2056989019008508

**Published:** 2019-06-25

**Authors:** Johannes Merkelbach, Walter Frank

**Affiliations:** a Institut für Anorganische Chemie und Strukturchemie, Lehrstuhl II: Material- und Strukturforschung, Heinrich-Heine-Universität Düsseldorf, Universitätsstrasse 1, D-40225 Düsseldorf, Germany

**Keywords:** Tin complexes, stannylenes, arene complexes, tin-arene π bonding, ring slippage, prehnitene complexes, tetra­chlorido­aluminates, tetra­chlorido­gallates, crystal structure

## Abstract

From solutions of prehnitene and the ternary halides (SnCl)[*M*Cl_4_] (*M* = Al, Ga) in chloro­benzene, the new cationic Sn^II^–π-arene complexes *catena*-poly[[chlorido­aluminate(III)]-tri-μ-chlorido-4′:1κ^2^ *Cl*,1:2κ^4^ *Cl*-[(η^6^-1,2,3,4-tetra­meth­yl­benzene)­tin(II)]-di-μ-chlorido-2:3κ^4^ *Cl*-[(η^6^-1,2,3,4-tetra­methyl­benzene)­tin(II)]-di-μ-chlorido-3:4κ^4^ *Cl*-[chlorido­aluminate(III)]-μ-chlorido-4:1′κ^2^ *Cl*], [Al_2_Sn_2_Cl_10_(C_10_H_14_)_2_]_*n*_, (**1**) and *catena*-poly[[chlorido­gallate(III)]-tri-μ-chlor­ido-4′:1κ^2^ *Cl*,1:2κ^4^ *Cl*-[(η^6^-1,2,3,4-tetra­methyl­benzene)­tin(II)]-di-μ-chlorido-2:3κ^4^ *Cl*-[(η^6^-1,2,3,4-tetra­methyl­benzene)­tin(II)]-di-μ-chlorido-3:4κ^4^ *Cl*-[chlor­ido­gallate(III)]-μ-chlorido-4:1′κ^2^ *Cl*], [Ga_2_Sn_2_Cl_10_(C_10_H_14_)_2_]_*n*_, (**2**), were isolated. In these first main-group metal–prehnitene complexes, the distorted η^6^ arene π-bonding to the tin atoms of the Sn_2_Cl_2_^2+^ moieties in the centre of [{1,2,3,4-(CH_3_)_4_C_6_H_2_}_2_Sn_2_Cl_2_][*M*Cl_4_]_2_ repeating units (site symmetry ) is characterized by: (i) a significant ring slippage of *ca* 0.4 Å indicated by the dispersion of Sn—C distances [**1**: 2.881 (2)–3.216 (2) Å; **2**: 2.891 (3)–3.214 (3) Å]; (ii) the non-methyl-substituted arene C atoms positioned closest to the Sn^II^ central atom; (iii) a pronounced tilt of the plane of the arene ligand against the plane of the central (Sn_2_Cl_2_)^2+^ four-membered ring species [**1**: 15.59 (11)°, **2**: 15.69 (9)°]; (iv) metal–arene bonding of medium strength as illustrated by application of the bond-valence method in an indirect manner, defining the π-arene bonding inter­action of the Sn^II^ central atoms as *s*(Sn^II^—arene) = 2 − Σ*s*(Sn^II^—Cl), that gives *s*(Sn^II^—arene) = 0.37 and 0.38 valence units for the aluminate and the gallate, respectively, indicating that comparatively strong main-group metal–arene bonding is present and in line with the expectation that [AlCl_4_]^−^ is the slightly weaker coordinating anion as compared to [GaCl_4_]^−^.

## Abstract 2

The first main-group-metal–prehnitene π complexes have been obtained in form of the isotypic pair {[{1,2,3,4-(CH_3_)_4_C_6_H_2_}_2_Sn_2_Cl_2_][*M*Cl_4_]_2_}_*x*_ (*M* = Al, Ga) and crystal structure determinations thereof give strong evidence that a distorted η^6^ coordination mode, characterized by a small but significant ring slippage of *ca* 0.4 Å as well as a pronounced tilt of the plane of the arene ligand against the plane of the central (Sn_2_Cl_2_)^2+^ four-membered ring species, is an intrinsic feature of this kind of arene complexed dimeric chlorido­stannylene cation. Application of the bond-valence method in a indirect manner yields empirical bond valences of 0.38 and 0.37, respectively, which allow for classifying the metal–π-arene bonding as a strong non-covalent inter­action, which is in line with the expectation that [AlCl_4_]^−^ is the slightly weaker coordinating anion as compared to [GaCl_4_]^−^.

## Chemical context   

Compounds that are known today to have arene (= benzen­oid) mol­ecules π-bonded to main-group metal central atoms have been studied since the late 19^th^ century (Smith, 1879 ▸; Smith & Davis, 1882 ▸; Lecoq de Boisbaudran, 1881 ▸). The best recognized work of the early period seems to be the series of investigations by Menshutkin, exploring the composition of compounds in systems of the type *EX*_3_/arene (*E* = As, Sb; *X* = Cl, Br), subsequently often referred to as ‘Menshutkin complexes’ (*e.g.* Menshutkin, 1911 ▸). However, the nature of bonding in such compounds remained unclear until the first structure determinations of *p*-block-metal–arene complexes were published in the late 1960s (Lüth & Amma, 1969 ▸; Hulme & Szymanski, 1969 ▸). Although a significant number of cationic main-group metal–π-arene complexes have been synthesized and structurally characterized since then (see review by Schmidbaur & Schier, 2008 ▸), the knowledge of isotypic pairs containing the same cation but different anions is so far limited to two couples of bis­(arene) complexes, *viz*. {[(C_6_H_6_)_2_Ga][Ga*X*_4_]}_2_ [*X* = Cl (Schmidbaur *et al.* 1983 ▸), Br (Uson-Finkenzeller *et al.*, 1986 ▸)] and {[(1,2,4-(CH_3_)_3_C_6_H_3_)_2_Tl][*M*Cl_4_]}_2_ (*M* = Al, Ga; Frank *et al.*, 1996 ▸). Only the latter pair was compared in detail.
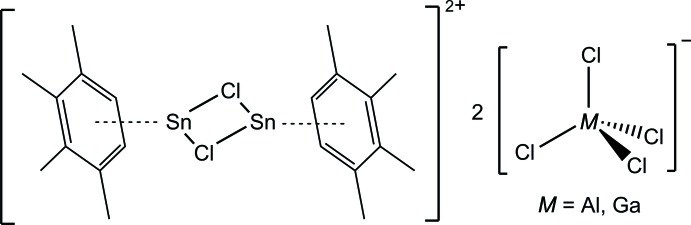


We herein describe the synthesis and structural investigation of {[{1,2,3,4-(CH_3_)_4_C_6_H_2_}_2_Sn_2_Cl_2_][*M*Cl_4_]_2_}_*x*_ [*M* = Al (**1**), Ga (**2**)], the first couple of isotypic mono(arene) complexes. In relation to previous work on structurally related compounds within the class [(arene)_2_Sn_2_Cl_2_][AlCl_4_]_2_ (Weininger *et al.*, 1979 ▸; Frank, 1990*a* ▸; Schmidbaur *et al.*, 1990 ▸; for further information see Section 4), a detailed analysis of the structural parameters of the isotypic cationic tin(II)–π-arene title complexes allows for: (i) identification of the intrinsic features of the π-bonding geometry of mono(arene) complexation in this class; (ii) investigation of the impact of anion change on the π-bonding situation unaffected by more principal structural differences; (iii) the indirect estimation of an empirical bond valence for the π-arene bonding as introduced to organometallic chemistry by one of us in the early 1990s (Frank, 1990*a* ▸,*b* ▸,*c* ▸). The title compounds are the first main-group metal–prehnitene π complexes. Strictly anhydrous conditions are needed for successful syntheses from the ternary halides Sn*M*Cl_5_ (= (SnCl)[*M*Cl_4_]; *M* = Al, Ga; Schloots & Frank, 2016 ▸) and prehnitene (1,2,3,4-tetra­methyl­benzene) in the inert solvent chloro­benzene and for the subsequent crystallization.

## Structural commentary   

**Figure 1:**
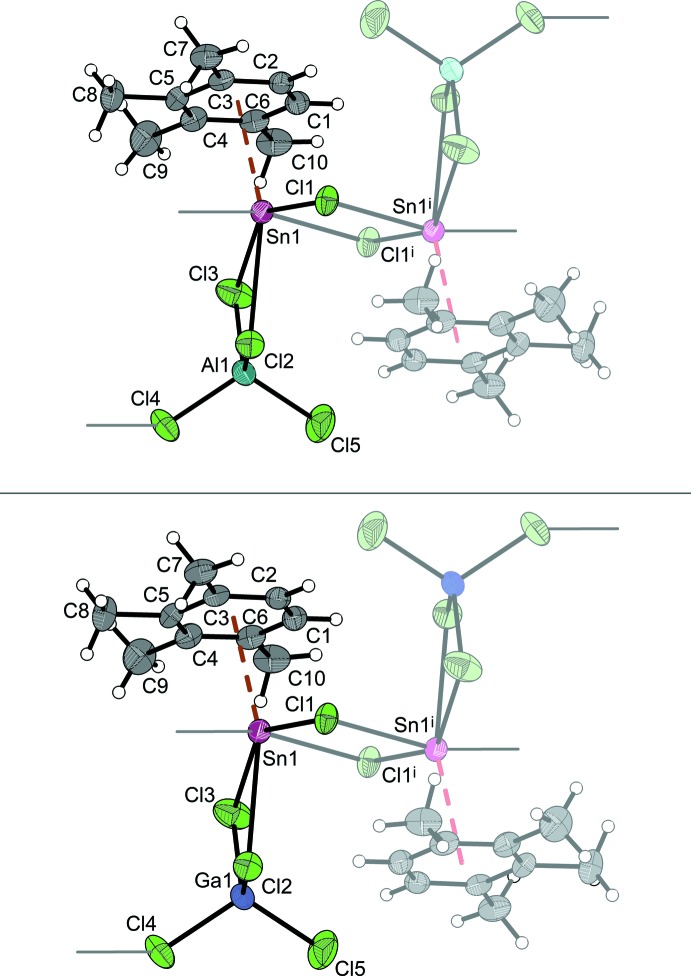
Asymmetric units of the crystal structures of **1** (top) and **2** (bottom) displaying the atom-labelling schemes, and in transparent mode the symmetry-related second half completing the dimeric building block that defines the repeating units of the coordination polymeric, secondary structure of the compounds[(symmetry code (i) 1 − *x*, −y, 1 − *z*]. The direction of secondary bonding to atoms of the neighbouring moieties is indicated by thin sticks. Displacement ellipsoids are drawn at the 50% probability level, hydrogen atoms are drawn with an arbitrary radius.

**Figure 2:**
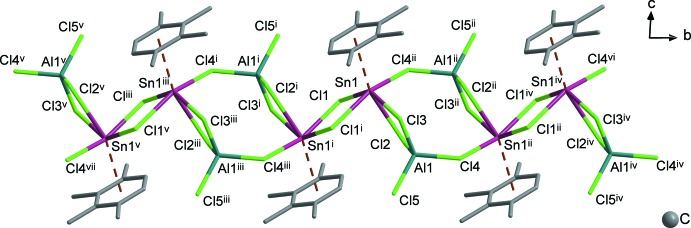
Coordination polymeric chain in the crystal of **1** [view direction [100]; symmetry codes: (i) 1 − *x*, −y, 1 − *z*; (ii) 1 − *x*, 1 − *y*, 1 − *z*; (iii) *x*, −1 + *y*, *z*; (iv) *x*, 1 + *y*, *z*; (v) 1 − *x*, −1 − *y*, 1 − *z*; (vi) 1 − *x*, 2 − *y*, 1 − *z*; (vii) *x*, − 2 + *y*, *z*]. Features indicative of the mode of concatenation of the characteristic building blocks are: (i) the parallel orientation of the Sn1⋯Sn1^i^ connecting line with respect to the chain building direction; (ii) the exclusively translational character of chain growth.

The asymmetric units of the isotypic compounds **1** and **2** consist of one half of a Sn_2_Cl_2_^2+^ moiety close to a centre of inversion, one [*M*Cl_4_]^−^ moiety and one prehnitene mol­ecule, all in general positions. As shown in Fig. 1 ▸, these components define one half of the centrosymmetric building block that represents the crystallographic repeating unit of a coordination-polymeric chain in which [{1,2,3,4-(CH_3_)_4_C_6_H_2_}_2_Sn_2_Cl_2_]^2+^ cations are connected by two [*M*Cl_4_]^−^ anions in a 1*κ* ^2^ *Cl*,*Cl* ^2^:3*κCl* ^3^-bridging mode. Bond lengths within the dimeric chlorido­stannylene cation (in direct comparison, Sn—Cl bond lengths and selected further geometric details of the bonding situation at the tin central atoms of **1** and **2** are given in Table 1 ▸) and the chlorido­metallate anions [*M*—Cl = 2.1058 (10)–2.1715 (9) Å (**1**) and 2.1439 (10)–2.2159 (10) Å (**2**); for almost undistorted anions see ortho­rhom­bic Li[AlCl_4_] (Prömper & Frank, 2017 ▸) and Ga[GaCl_4_] (Schmidbaur *et al.*, 1987 ▸)] are as expected, taking into account the mode of association of these species in the polymeric chains. For **1**, a section of this chain involving three repeating units is displayed in Fig. 2 ▸. Considering the dimensions of the repeating unit along the chain concatenation direction [010] *and* the orientation of the Sn⋯Sn^i^ connection line with respect to this direction, the secondary structure of **1** and **2** established by the mode of concatenation differs principally from all other related structures apart from that of the mesitylene derivative. However, for this derivative the tertiary structure established by the arrangement of columns is entirely different. A more detailed discussion of the packing is given in Section 3.

**Figure 3:**
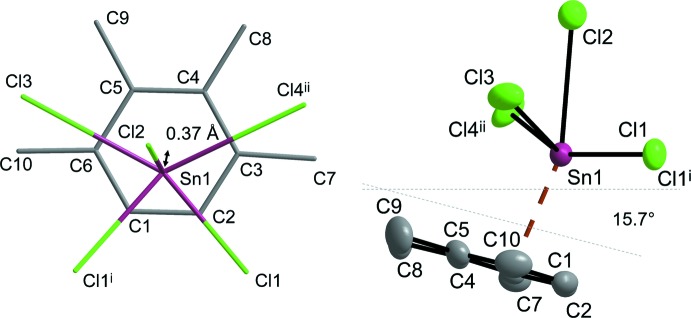
Sn^II^ coordination environment of **1** illustrating the ring slippage (left) and the arene tilt angle (right; displacement ellipsoids drawn at 50% probability level). Symmetry codes: (i) 1 − *x*, −y, 1 − *z*; (ii) 1 − *x*, 1 − *y*, 1 − *z*.

Two primary and three secondary bonded chlorine atoms of the dimeric cation and the metallate anions, respectively, as well as one π-coordinating prehnitene mol­ecule establish the coordination sphere around the Sn^II^ central atom (Fig. 3 ▸). Considering the arene π-ligand as occupying one coordination site only, the coordination number is six. The range of *cis*-Cl—Sn—Cl angles [**1**: 66.360 (18)–120.01 (2)°; **2**: 67.82 (2)–121.37 (3)°] is far from allowing the coordination to be described as octa­hedral, and in our feeling a description as *ψ*-penta­gonal bipyramidal with the arene ligand and Cl2 in axial position [Cnt_arene_—Sn—Cl 160.856 (15)° (**1**) and 161.137 (18)° (**2**)] and with the probable equatorial position of the stereochemically active 5*s* ^2^ lone pair between Cl3 and Cl4^ii^ [Cl3—Sn—Cl4^ii^ 120.01 (2)° (**1**) and 121.37 (3)° (**2**)] is much more appropriate. This fits to the observation that the best plane of the arene atoms C1 to C6 at this side of the coordination sphere is more tilted away from this probable lone pair position in the plane defined by Sn1, Cl3 and Cl4^ii^ [27.50 (8)° (**1**) and 26.98 (8)° (**2**)] than from the equatorial ligands Cl1 and Cl1^i^ at the opposite side [**1**: 15.59 (11)°, **2**: 15.69 (9)°]. As documented in the two sections of Fig. 3 ▸, the tin–π-prehnitene bonding is characterized by the non-methyl-substituted arene C atoms positioned closest to the Sn^II^ central atom, by a significant ring slippage (**1** and **2**: 0.37 Å) also indicated by the dispersion of Sn—C distances [**1**: 2.881 (2)–3.216 (2) Å; **2**: 2.891 (3)–3.214 (3) Å], and by the tilt of the plane of the arene ligand against the plane of the central planar (Sn_2_Cl_2_)^2+^ four-membered ring species as mentioned above. Finally, in the absence of – the transition-metal-specific – π-arene backbonding, it is not unexpected that no significant influence of the Sn^II^ coordination on the prehnitene six-membered ring geometry is found in comparison with the results of DFT calculations (Becke, 1993 ▸) for non-coordinating prehnitene (Table 1 ▸).

The π-bonding inter­action in **1** and **2** is of medium strength on the overall scale including all types of arene π-bonding, but strong on the scale of non-covalent main-group metal–arene bonding, as easily illustrated by the application of the bond-valence method according to the formalism of Brown (2009 ▸) in an indirect manner: defining the bond valence of the π-arene bonding to the Sn^II^ central atom as *s*(Sn^II^—arene) = 2 − Σ*s*(Sn^II^—Cl) (Frank, 1990*a* ▸), which gives *s*(Sn^II^—arene) = 0.37 and 0.38 valence units for the aluminate and the gallate, respectively. These values are in line with the expectation that [AlCl_4_]^−^ is the slightly weaker coordinating anion as compared to [GaCl_4_]^−^. A more detailed analysis of *M*—Cl, Sn—Cl and Sn—Cnt_arene_ distances shows that the anion change does not have impact on the bonding within the (Sn_2_Cl_2_)^2+^ moiety, but a small but significant influence can be traced along a path of bonding from the central atom *M*1 of the anion to the arene ligand [Al1(Ga1)—Cl2—Sn1—Lsqpl_arene_ [**1**: 2.1715 (9), 3.0340 (7), 2.6898 (11) Å; **2**: 2.2159 (9), 3.0155 (9), 2.6997 (14) Å)]. Fully consistent with the observation that the Sn^II^— arene distance is shorter in the aluminate, the distance to the *trans*-ligand Cl2 is longer and Al1—Cl2 (= Al1—Cl_mean_ + 1.8%) is *relatively* shorter than Ga1—Cl2 (= Ga1—Cl_mean_ + 1.95%). In both **1** and **2**, the tin–arene bonding is remarkably stronger than the bonding to the Cl2 ligand in the *trans*-position [*s*(Sn—Cl2) = 0.21 (1) and 0.22 (2) valence units]. Inter­estingly, as documented by the dispersion of Bi—C distances [2.753 (9)–3.214 (9) Å] and the arene tilt angle against the plane defined by the Bi and the two primarily bonded Cl atoms in the BiCl_2_^+^ moiety (20.6°), the Bi^III^ coordination geometry in the monocationic (mono)hexa­methyl­benzene bis­muth complex {[((CH_3_)_6_C_6_)BiCl_2_][AlCl_4_]}_2_ (Frank *et al.*, 1987 ▸) is closely related to the Sn^II^ coordination sphere of **1** and **2**.

## Supra­molecular features   

**Figure 4:**
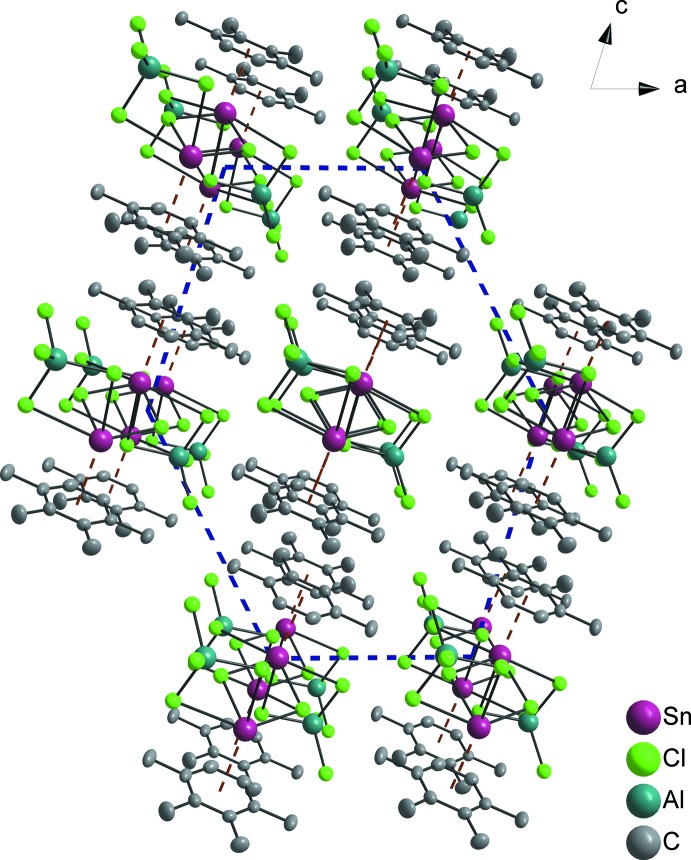
Distorted hexa­gonal packing of chains in the crystal of **1** (view direction [0
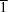
0]). The most characteristic feature is the parallel orientation of the planes of neighbouring prehnitene ligands in the [001] direction.

**Figure 5:**
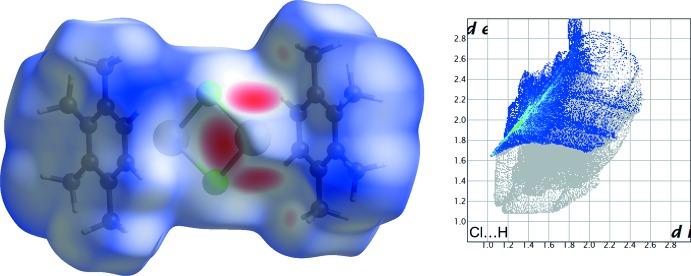
Three-dimensional Hirshfeld (*d*_norm_) surface for the [(1,2,3,4-tetra­methyl­benzene)_2_Sn_2_Cl_2_]^2+^ moiety of **1** (left) and two-dimensional fingerprint plot for the H⋯Cl contacts (right); prepared using *CrystalExplorer17.5* (Turner *et al.*, 2017 ▸). The characteristic feature in the fingerprint plot mainly corresponds to the contacts C10—H103⋯Cl3 {C—H 0.97 Å, H⋯Cl 2.79 [2.74] Å, C—H⋯Cl 146.3 [149.4]°, C⋯Cl 3.633 (3) [3.605 (4)] Å} and C7—H71⋯Cl4^ii^ {C—H 0.97 Å, H⋯Cl 2.80 [2.78] Å, C—H⋯Cl 143.6 [144.1]°, C⋯Cl 3.622 (3) [3.612 (4)] Å}; values for **2** given in square brackets.

As in all {[(arene)_2_Sn_2_Cl_2_][AlCl_4_]_2_}_*x*_ structures described before [arene = benzene, toluene (two polymorphs), *p*-xylene, mesitylene (see Section 4 and for a detailed comparison; Frank, 1990*a* ▸), in both **1** and **2** the chains (propagating along [010]) are aligned parallel to each other, resulting in a distorted hexa­gonal packing of rods. However, taking into account primary, secondary *and* tertiary bonding, the crystal structure of **1** and **2** is unique. Exemplarily, Fig. 4 ▸ shows the packing of **1**, mainly characterized by the face-to-face orientation of the prehnitene ligands of neighbouring columns in direction [001]. The orientation of the arene mol­ecules arranged parallel to each other suggests the presence of π–π inter­actions. However, the distance between the best planes of the prehnitene ligands in discussion is greater than 3.6 Å and only ‘conventional’ van der Waals inter­actions have to be assumed in this direction. A Hirshfeld analysis of the [(1,2,3,4-tetra­methyl­benzene)_2_Sn_2_Cl_2_]^2+^ moiety (Fig. 5 ▸) clearly shows three contact points between (Sn_2_Cl_2_)^2+^ cations and [*M*Cl­_4_]^−^ anions as described above. Additionally, it reveals a weak C—H⋯Cl inter­action between the methyl groups in the 1- and 4-positions of the prehnitene ligand and chlorine atoms of the [*M*Cl­_4_]^−^ anions (Tables 2 ▸ and 3 ▸), as shown in the corres­ponding fingerprint plot.

## Database survey   

A search of the Cambridge Structural Database (Version 5.40, update November 2018; Groom *et al.*, 2016 ▸) for tin(II) complexes with arene (benzenoid) ligands, displaying at least three bonds of type ‘any’ between the tin central atom and carbon atoms of the arene moiety, resulted in 15 hits, including SPHOSN (Lefferts *et al.*, 1980 ▸) with η^6^ but extremely weak intra­molecular bonding to the phenyl group of a di­thio­phosphate ligand. Because some of the π complexes known to the authors are missed by this search strategy, in addition a search for structures displaying at least three Sn⋯C non-bonded contacts shorter than 3.67 Å (equal to the sum of van der Waals radii (3.87 Å) minus 0.2 Å) was performed and gave an additional 26 hits. However, all but six of these are associated with very weak and/or strongly distorted intra- or inter­molecular contacts to phenyl or phenyl­ene groups within one mol­ecule or between same mol­ecules in the solid. Of the 15 + 6 structures identified by this dual search strategy, six (BENZSN10, Rodesiler *et al.*, 1975 ▸; IZUXAD, Schäfer *et al.*, 2011 ▸; JAVJIZ, Schmidbaur *et al.*,1989*c* ▸; KIKDIR, Probst *et al.*, 1990 ▸; ZEMFAB and ZEMFEF, Schleep *et al.*, 2017*a* ▸) have ‘dicationic’ Sn^II^ central species. Comparatively weak bonding of benzene mol­ecules to the Sn^II^ central atoms is given in the benzene-solvated mixed-valence Sn^II^/Sn^IV^ oxido-tri­fluoro­acetate OFACSO (Birchall & Johnson, 1981 ▸). HOQYIX (Beckmann *et al.*, 2012 ▸) is a bis­(arene) complex of Cp*Sn^+^ involving two phenyl groups of the [BPh_4_]^−^ counter-ion, while YAWNOC is a perfluoro­alk­oxy­aluminate containing the [CpSn(C_6_H_5_Me)]^+^ cation (Schleep *et al.*, 2017*b* ▸). ZEMFIJ contains the mesitylene-complexed dimeric bromido­stannylene cation (Sn_2_Br_2_)^2+^ (Schleep *et al.*, 2017*a* ▸). Of the remaining ten structures, all containing the dimeric chlorido­stannylene cation (Sn_2_Cl_2)_^2+^, one is a bis­(arene)chlorido­tin(II) tetra­chlorido­aluminate (VAWCAX Schmidbaur *et al.*, 1989*b* ▸), one a mono(arene)chlorido­tin(II) tetra­chlorido­gallate (JENMEU; Frank, 1990*b* ▸) and eight are mono(arene)chlorido­tin(II) tetra­chlorido­aluminates, including the triptycene complex VOGXEU (Schmidbaur *et al.*, 1991 ▸), the benzene complex CBZSNA10 (Weininger *et al.*, 1979 ▸), the polymorphic toluene complexes VEXHOV and VEXHOV01 (Frank, 1990*a* ▸), the *p*-xylene complex CPXSNA10 (Weininger *et al.*, 1979 ▸), the mesitylene complex SESSOY (Schmidbaur *et al.*, 1990 ▸) and SESSOY01 (Frank, 1990*a* ▸) and the hexa­methyl­benzene complex SANMUP (Schmidbaur *et al.*, 1989*a* ▸). Like the title structures, the benzene, toluene, *p*-xylene and mesitylene complexes are coordination polymers with bridging [AlCl_4_]^−^ anions; however, none of these is in a homotypic relationship to the title structures or to one of the others. Considering arene complexes of *p*-block elements in general, there is only one AlCl_4_^−^/GaCl_4_^−^-isotypic pair of compounds known, *viz*. the bis­(arene)thallium tetra­halogenidometallates ZOFGEG and ZOFGAC (Frank *et al.*, 1996 ▸). Finally, **1** and **2** are the first main-group metal–prehnitene complexes.

## Synthesis and crystallization   

Synthesis and crystallization of **1** and **2** were carried out under an argon atmosphere applying strictly anhydrous conditions using a glass vacuum line equipped with J. Young high-vacuum PTFE valves. Gallium trichloride was used as purchased (Sigma Aldrich, 99.999%), aluminum trichloride (Sigma Aldrich, 99.99%) was purified by repeated sublimation, SnCl_2_ (Acros Organics, 98%) was dried with acetic anhydride, the prehnitene/chloro­benzene (Alfa Aesar, 95%; Acros Organics, 99+ %) mixture purified and dried through an alumina packed column. Both **1** and **2** can be obtained using the ternary halide [SnCl][*M*Cl_4_] (*M* = Al, Ga) directly (Schloots & Frank, 2016 ▸) or using a SnCl_2_/*M*Cl_3_ mixture instead.

40 mg; 0.12 mmol (160 mg; 0.44 mmol) of [SnCl][AlCl_4_] ([SnCl][GaCl_4_]) were dissolved in 4 ml of a prehnitene-chloro­benzene mixture (1.3 mmol to 37.6 mmol) at 343 K. Colourless needles of **1** and **2** were obtained by slowly cooling the solution to room temperature in qu­anti­tative yield.

**[{1,2,3,4-(CH_3_)_4_C_6_H_2_}_2_Sn_2_Cl_2_][AlCl_4_]_2_ (1)**: Raman (cm^−1^): 3060 *ν*(C_ar_—H), 2933 *ν*(CH_3_), 1581 *ν*(C C), 1388 *δ*(CH_3_), 1247 and 640 *δ*(C C—H), 347 *ν*(AlCl_4_^−^), 242 *δ*(Sn_2_Cl_2_­^2+^), 121 *δ*(AlCl_4_^−^). Elemental analysis (calculated): C, 25.93 (26.25); H, 3.06 (3.06) %. M.p. (decomp.) 432 K.

**[{1,2,3,4-(CH_3_)_4_C_6_H_2_}_2_Sn_2_Cl_2_][GaCl_4_]_2_ (2):** Raman (cm^−1^): 3057 *ν*(C_ar_—H), 2930 *ν*(CH_3_), 1580 *ν*(C C), 1388 *δ*(CH_3_), 1247 and 640 *δ*(C C—H), 348 *ν*(GaCl_4_^−^), 241 *δ*(Sn_2_Cl_2_­^2+^), 115 *δ*(GaCl_4_^−^). Elemental analysis (calculated): C, 24.03 (24.00); H, 2.84 (2.80) %. M.p. (decomp.) 425 K.

## Refinement   

Crystal data, data collection and structure refinement details are summarized in Table 4 ▸. The positions of all hydrogen atoms were identified *via* subsequent difference-Fourier syntheses. In the refinement a riding model was applied using idealized C—H bond lengths [0.94 (CH) and 0.97 (CH_3_) Å] as well as H—C—H and C—C—H angles. In addition, the H atoms of the CH_3_ groups were allowed to rotate around the neighbouring C—C bonds. The *U*_iso_ values were set to 1.5*U*_eq_(C_meth­yl_) and 1.2*U*_eq_(C_ar_).

## Supplementary Material

Crystal structure: contains datablock(s) I, II, global. DOI: 10.1107/S2056989019008508/wm5510sup1.cif

Structure factors: contains datablock(s) I. DOI: 10.1107/S2056989019008508/wm5510Isup2.hkl

Structure factors: contains datablock(s) II. DOI: 10.1107/S2056989019008508/wm5510IIsup3.hkl

CCDC references: 1923088, 1923087

Additional supporting information:  crystallographic information; 3D view; checkCIF report

## Figures and Tables

**Table 1:** Selected bond lengths and contact distances (Å) in **1** and **2** and corresponding ring slippage values and bond valences, calculated using the Brown formalism (Brown, 2009 ▸) with *r*_0_ = 2.42 and *B* = 0.39 (Frank, 1990*a* ▸). Cnt_arene_ = arene centre; Lsqpl_arene_ = arene plane. C C bond lengths were calculated on the B3LYP/6–311++G(d,p) level of theory using the *GAUSSIAN09* program package (Frisch *et al.*, 2009 ▸).

	**1**	**2**			**1**	**2**
Sn1—C1	2.881 (2)	2.891 (3)		Sn1—Cl1	2.6316 (6)	2.6299 (8)
Sn1—C2	2.915 (2)	2.921 (3)		Sn1—Cl1^i^	2.6425 (6)	2.6481 (7)
Sn1—C3	3.097 (2)	3.104 (3)		Sn1—Cl2	3.0340 (7)	3.0155 (9)
Sn1—C4	3.216 (2)	3.214 (3)		Sn1—Cl3	3.2432 (8)	3.2597 (11)
Sn1—C5	3.181 (2)	3.185 (3)		Sn1—Cl4^ii^	3.1722 (7)	3.1499 (9)
Sn1—C6	3.028 (2)	3.043 (3)				
						
	**1**	**2**	calculated		**1**	**2**
C1—C2	1.386 (4)	1.379 (5)	1.3888	*d*(Sn—Cnt_arene_)	2.716 (2)	2.725 (3)
C2—C3	1.394 (3)	1.396 (4)	1.3957	*d*(Sn—Lsqpl_arene_)	2.6898 (11)	2.6997 (14)
C3—C4	1.405 (3)	1.408 (4)	1.4082	Ring slippage	0.37	0.37
C4—C5	1.411 (3)	1.408 (5)	1.4112			
C5—C6	1.404 (4)	1.404 (5)	1.4082	Σ*s*(Sn—Cl)	1.62	1.63
C6—C1	1.393 (4)	1.388 (5)	1.3957	*s*(Sn—arene)	0.38	0.37

Symmetry codes: (i) 1 − *x*, −*y*, 1 − *z*; (ii) 1 − *x*, 1 − *y*, 1 − *z*.

**Table 2:** Hydrogen-bond geometry (Å, °) for **1** [Chem scheme1]

*D*—H⋯*A*	*D*—H	H⋯*A*	*D*⋯*A*	*D*—H⋯*A*
C10—H103⋯Cl3	0.97	2.79	3.633 (3)	146
C7—H71⋯Cl4^i^	0.97	2.80	3.622 (3)	144

Symmetry code: (i) 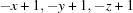.

**Table 3:** Hydrogen-bond geometry (Å, °) for **2** [Chem scheme1]

*D*—H⋯*A*	*D*—H	H⋯*A*	*D*⋯*A*	*D*—H⋯*A*
C10—H103⋯Cl3	0.97	2.74	3.605 (4)	149
C7—H71⋯Cl4^i^	0.97	2.78	3.612 (4)	144

Symmetry code: (i) 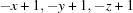.

**Table 4:** Experimental details

	**1**	**2**
Crystal data		
Chemical formula	[Al_2_Sn_2_Cl_10_(C_10_H_14_)_2_]	[Ga_2_Sn_2_Cl_10_(C_10_H_14_)_2_]
*M*_r_	914.30	999.78
Crystal system, space group	Triclinic, *P* 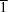	Triclinic, *P* 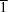
Temperature (K)	213	213
*a*, *b*, *c* (Å)	8.7512 (5), 9.1357 (6), 11.2803 (7)	8.7572 (4), 9.1310 (4), 11.2966 (5)
α, β, γ (°)	85.524 (5), 72.769 (5), 86.926 (5)	85.424 (3), 72.805 (3), 86.886 (4)
*V* (Å^3^)	858.30 (9)	859.73 (7)
*Z*	1	1
Radiation type	Mo *K*α	Mo *K*α
μ (mm^−1^)	2.30	3.77
Crystal size (mm)	0.27 × 0.17 × 0.13	0.61 × 0.13 × 0.03

Data collection		
Diffractometer	Stoe IPDS 2T	Stoe IPDS 2
Absorption correction	Multi-scan (Blessing, 1995 ▸)	Multi-scan (Blessing, 1995 ▸)
*T*_min_, *T*_max_	0.476, 0.697	0.376, 0.656
No. of measured, independent and observed [*I* > 2σ(*I*)] reflections	17750, 4585, 4326	17522, 4638, 4242
*R*_int_	0.040	0.048
(sin θ/λ)_max_ (Å^−1^)	0.686	0.686

Refinement		
*R*[*F* ^2^ > 2σ(*F* ^2^)], *wR*(*F* ^2^), *S*	0.028, 0.058, 1.24	0.033, 0.069, 1.22
No. of reflections	4585	4638
No. of parameters	158	158
H-atom treatment	H-atom parameters constrained	H-atom parameters constrained
Δρ_max_, Δρ_min_ (e Å^−3^)	0.59, −0.46	0.71, −0.50

Computer programs: *X-AREA* (Stoe & Cie, 2009 ▸), *SHELXT2014/5* (Sheldrick, 2015*a* ▸), *SHELXL2018/3* (Sheldrick, 2015*b* ▸), *DIAMOND* (Brandenburg, 2018 ▸) and *publCIF* (Westrip, 2010 ▸).

## supplementary crystallographic information

### catena-Poly[[chloridoaluminate(III)]-tri-µ-chlorido-4':1κ2Cl,1:2κ4Cl-[(η6-1,2,3,4-tetramethylbenzene)tin(II)]-di-µ-chlorido-2:3κ4Cl-[(η6-1,2,3,4-tetramethylbenzene)tin(II)]-di-µ-chlorido-3:4κ4Cl-[chloridoaluminate(III)]-µ-chlorido-4:1'κ2Cl] (I) Crystal data

**Table:**

[Al_2_Sn_2_Cl_10_(C_10_H_14_)_2_]	*Z* = 1
*M**_r_* = 914.30	*F*(000) = 444
Triclinic, *P*1	*D*_x_ = 1.769 Mg m^−^^3^
*a* = 8.7512 (5) Å	Mo *K*α radiation, λ = 0.71073 Å
*b* = 9.1357 (6) Å	Cell parameters from 24404 reflections
*c* = 11.2803 (7) Å	θ = 4.5–59.3°
α = 85.524 (5)°	µ = 2.30 mm^−^^1^
β = 72.769 (5)°	*T* = 213 K
γ = 86.926 (5)°	Needle, colorless
*V* = 858.30 (9) Å^3^	0.27 × 0.17 × 0.13 mm

### catena-Poly[[chloridoaluminate(III)]-tri-µ-chlorido-4':1κ2Cl,1:2κ4Cl-[(η6-1,2,3,4-tetramethylbenzene)tin(II)]-di-µ-chlorido-2:3κ4Cl-[(η6-1,2,3,4-tetramethylbenzene)tin(II)]-di-µ-chlorido-3:4κ4Cl-[chloridoaluminate(III)]-µ-chlorido-4:1'κ2Cl] (I) Data collection

**Table:**

Stoe IPDS 2T diffractometer	4326 reflections with *I* > 2σ(*I*)
Radiation source: fine-focus sealed tube	*R*_int_ = 0.040
ω scans	θ_max_ = 29.2°, θ_min_ = 2.2°
Absorption correction: multi-scan (Blessing, 1995)	*h* = −11→11
*T*_min_ = 0.476, *T*_max_ = 0.697	*k* = −12→12
17750 measured reflections	*l* = −15→15
4585 independent reflections	

### catena-Poly[[chloridoaluminate(III)]-tri-µ-chlorido-4':1κ2Cl,1:2κ4Cl-[(η6-1,2,3,4-tetramethylbenzene)tin(II)]-di-µ-chlorido-2:3κ4Cl-[(η6-1,2,3,4-tetramethylbenzene)tin(II)]-di-µ-chlorido-3:4κ4Cl-[chloridoaluminate(III)]-µ-chlorido-4:1'κ2Cl] (I) Refinement

**Table:**

Refinement on *F*^2^	Primary atom site location: structure-invariant direct methods
Least-squares matrix: full	Hydrogen site location: inferred from neighbouring sites
*R*[*F*^2^ > 2σ(*F*^2^)] = 0.028	H-atom parameters constrained
*wR*(*F*^2^) = 0.058	*w* = 1/[σ^2^(*F*_o_^2^) + (0.0163*P*)^2^ + 0.5546*P*] where *P* = (*F*_o_^2^ + 2*F*_c_^2^)/3
*S* = 1.24	(Δ/σ)_max_ = 0.001
4585 reflections	Δρ_max_ = 0.59 e Å^−^^3^
158 parameters	Δρ_min_ = −0.46 e Å^−^^3^
0 restraints	

### catena-Poly[[chloridoaluminate(III)]-tri-µ-chlorido-4':1κ2Cl,1:2κ4Cl-[(η6-1,2,3,4-tetramethylbenzene)tin(II)]-di-µ-chlorido-2:3κ4Cl-[(η6-1,2,3,4-tetramethylbenzene)tin(II)]-di-µ-chlorido-3:4κ4Cl-[chloridoaluminate(III)]-µ-chlorido-4:1'κ2Cl] (I) Special details

**Table:**

Geometry. All esds (except the esd in the dihedral angle between two l.s. planes) are estimated using the full covariance matrix. The cell esds are taken into account individually in the estimation of esds in distances, angles and torsion angles; correlations between esds in cell parameters are only used when they are defined by crystal symmetry. An approximate (isotropic) treatment of cell esds is used for estimating esds involving l.s. planes.

### catena-Poly[[chloridoaluminate(III)]-tri-µ-chlorido-4':1κ2Cl,1:2κ4Cl-[(η6-1,2,3,4-tetramethylbenzene)tin(II)]-di-µ-chlorido-2:3κ4Cl-[(η6-1,2,3,4-tetramethylbenzene)tin(II)]-di-µ-chlorido-3:4κ4Cl-[chloridoaluminate(III)]-µ-chlorido-4:1'κ2Cl] (I) Fractional atomic coordinates and isotropic or equivalent isotropic displacement parameters (Å^2^)

**Table:**

	*x*	*y*	*z*	*U*_iso_*/*U*_eq_	
Sn1	0.52308 (2)	0.16549 (2)	0.60211 (2)	0.02833 (5)	
Cl1	0.29871 (6)	0.01611 (6)	0.55845 (5)	0.03375 (12)	
Cl2	0.52884 (7)	0.36374 (7)	0.37225 (6)	0.03974 (13)	
Cl3	0.85918 (10)	0.32842 (9)	0.47942 (8)	0.05591 (19)	
Cl4	0.80050 (8)	0.64593 (6)	0.30806 (7)	0.04703 (16)	
Cl5	0.90510 (11)	0.32035 (10)	0.15790 (8)	0.0646 (2)	
Al1	0.78043 (9)	0.41345 (7)	0.32595 (7)	0.03229 (15)	
C1	0.6234 (3)	−0.0503 (3)	0.7664 (2)	0.0366 (5)	
H1	0.671568	−0.133161	0.723925	0.044*	
C2	0.4588 (3)	−0.0430 (3)	0.8189 (2)	0.0335 (5)	
H2	0.397040	−0.120733	0.811111	0.040*	
C3	0.3833 (3)	0.0780 (3)	0.8831 (2)	0.0305 (4)	
C4	0.4771 (3)	0.1937 (3)	0.8931 (2)	0.0330 (5)	
C5	0.6446 (3)	0.1861 (3)	0.8393 (2)	0.0349 (5)	
C6	0.7188 (3)	0.0631 (3)	0.7757 (2)	0.0358 (5)	
C7	0.2040 (3)	0.0788 (3)	0.9401 (3)	0.0434 (6)	
H71	0.155733	0.161604	0.903607	0.065*	
H72	0.178309	0.087089	1.029158	0.065*	
H73	0.162684	−0.011828	0.924343	0.065*	
C8	0.3978 (4)	0.3254 (3)	0.9619 (3)	0.0501 (7)	
H81	0.418526	0.412853	0.905839	0.075*	
H82	0.440483	0.336065	1.030844	0.075*	
H83	0.283293	0.311956	0.993471	0.075*	
C9	0.7448 (4)	0.3112 (4)	0.8510 (3)	0.0588 (8)	
H91	0.855725	0.292126	0.804884	0.088*	
H92	0.736307	0.319407	0.937973	0.088*	
H93	0.706411	0.402187	0.817729	0.088*	
C10	0.8978 (3)	0.0490 (4)	0.7171 (3)	0.0501 (7)	
H101	0.923939	−0.041956	0.675420	0.075*	
H102	0.951224	0.048501	0.781249	0.075*	
H103	0.933184	0.131365	0.657192	0.075*	

### catena-Poly[[chloridoaluminate(III)]-tri-µ-chlorido-4':1κ2Cl,1:2κ4Cl-[(η6-1,2,3,4-tetramethylbenzene)tin(II)]-di-µ-chlorido-2:3κ4Cl-[(η6-1,2,3,4-tetramethylbenzene)tin(II)]-di-µ-chlorido-3:4κ4Cl-[chloridoaluminate(III)]-µ-chlorido-4:1'κ2Cl] (I) Atomic displacement parameters (Å^2^)

**Table:**

	*U*^11^	*U*^22^	*U*^33^	*U*^12^	*U*^13^	*U*^23^
Sn1	0.02904 (8)	0.02763 (8)	0.02706 (7)	−0.00218 (5)	−0.00572 (5)	−0.00324 (5)
Cl1	0.0225 (2)	0.0373 (3)	0.0385 (3)	−0.00117 (19)	−0.0019 (2)	−0.0127 (2)
Cl2	0.0330 (3)	0.0419 (3)	0.0428 (3)	−0.0057 (2)	−0.0084 (2)	−0.0019 (2)
Cl3	0.0568 (4)	0.0534 (4)	0.0656 (5)	−0.0169 (3)	−0.0332 (4)	0.0179 (3)
Cl4	0.0361 (3)	0.0256 (3)	0.0687 (4)	−0.0010 (2)	−0.0004 (3)	0.0026 (3)
Cl5	0.0581 (5)	0.0649 (5)	0.0572 (5)	0.0054 (4)	0.0080 (4)	−0.0241 (4)
Al1	0.0307 (3)	0.0257 (3)	0.0363 (4)	−0.0004 (3)	−0.0037 (3)	−0.0012 (3)
C1	0.0403 (13)	0.0347 (12)	0.0313 (11)	0.0071 (10)	−0.0066 (10)	−0.0016 (9)
C2	0.0368 (12)	0.0333 (11)	0.0307 (11)	−0.0060 (9)	−0.0102 (9)	0.0015 (9)
C3	0.0265 (10)	0.0389 (12)	0.0241 (10)	−0.0032 (9)	−0.0045 (8)	0.0007 (8)
C4	0.0337 (12)	0.0373 (12)	0.0279 (10)	−0.0004 (9)	−0.0079 (9)	−0.0066 (9)
C5	0.0300 (11)	0.0446 (13)	0.0318 (11)	−0.0072 (9)	−0.0103 (9)	−0.0030 (9)
C6	0.0261 (11)	0.0484 (14)	0.0308 (11)	0.0017 (9)	−0.0071 (9)	0.0034 (9)
C7	0.0281 (12)	0.0562 (16)	0.0408 (13)	−0.0037 (11)	−0.0039 (10)	0.0051 (11)
C8	0.0533 (17)	0.0464 (15)	0.0481 (16)	0.0030 (12)	−0.0075 (13)	−0.0196 (12)
C9	0.0485 (17)	0.067 (2)	0.065 (2)	−0.0225 (15)	−0.0179 (15)	−0.0148 (16)
C10	0.0274 (12)	0.0668 (19)	0.0498 (16)	0.0056 (12)	−0.0063 (11)	0.0099 (13)

### catena-Poly[[chloridoaluminate(III)]-tri-µ-chlorido-4':1κ2Cl,1:2κ4Cl-[(η6-1,2,3,4-tetramethylbenzene)tin(II)]-di-µ-chlorido-2:3κ4Cl-[(η6-1,2,3,4-tetramethylbenzene)tin(II)]-di-µ-chlorido-3:4κ4Cl-[chloridoaluminate(III)]-µ-chlorido-4:1'κ2Cl] (I) Geometric parameters (Å, º)

**Table:**

Sn1—Cl1	2.6316 (6)	C2—H2	0.9400
Sn1—Cl1^i^	2.6425 (6)	C3—C4	1.405 (3)
Sn1—Cl2	3.0340 (7)	C3—C7	1.509 (3)
Sn1—Cl3	3.2432 (8)	C4—C5	1.411 (3)
Sn1—Cl4^ii^	3.1722 (7)	C4—C8	1.506 (3)
Sn1—C1	2.881 (2)	C5—C6	1.404 (4)
Sn1—C2	2.915 (2)	C5—C9	1.513 (4)
Sn1—C3	3.097 (2)	C6—C10	1.512 (3)
Sn1—C4	3.216 (2)	C7—H71	0.9700
Sn1—C5	3.181 (2)	C7—H72	0.9700
Sn1—C6	3.028 (2)	C7—H73	0.9700
Sn1—Al1	3.8983 (8)	C8—H81	0.9700
Sn1—Sn1^i^	4.0476 (4)	C8—H82	0.9700
Al1—Cl2	2.1715 (9)	C8—H83	0.9700
Al1—Cl3	2.1249 (11)	C9—H91	0.9700
Al1—Cl4	2.1294 (9)	C9—H92	0.9700
Al1—Cl5	2.1058 (10)	C9—H93	0.9700
C1—C2	1.386 (4)	C10—H101	0.9700
C1—C6	1.393 (4)	C10—H102	0.9700
C1—H1	0.9400	C10—H103	0.9700
C2—C3	1.394 (3)		
			
Cl1—Sn1—Cl1^i^	79.753 (18)	Al1—Cl4—Sn1^ii^	116.50 (3)
Cl1—Sn1—Cl2	88.426 (19)	Cl5—Al1—Cl3	113.35 (5)
Cl1—Sn1—Cl3	145.46 (2)	Cl5—Al1—Cl4	110.64 (5)
Cl1—Sn1—Cl4^ii^	73.398 (18)	Cl3—Al1—Cl4	108.81 (5)
Cl1^i^—Sn1—Cl2	84.476 (19)	Cl5—Al1—Cl2	109.07 (5)
Cl1^i^—Sn1—Cl3	74.86 (2)	Cl3—Al1—Cl2	106.38 (4)
Cl1^i^—Sn1—Cl4^ii^	147.90 (2)	Cl4—Al1—Cl2	108.40 (4)
Cl2—Sn1—Cl3	66.360 (18)	C2—C1—C6	121.1 (2)
Cl2—Sn1—Cl4^ii^	77.616 (19)	C2—C1—H1	119.4
Cl3—Sn1—Cl4^ii^	120.01 (2)	C6—C1—H1	119.4
Cl1—Sn1—C1	98.59 (6)	C1—C2—C3	121.0 (2)
Cl1—Sn1—C2	80.90 (5)	C1—C2—H2	119.5
Cl1—Sn1—C3	89.06 (4)	C3—C2—H2	119.5
Cl1—Sn1—C4	113.59 (5)	C2—C3—C4	118.8 (2)
Cl1—Sn1—C5	133.54 (5)	C2—C3—C7	118.7 (2)
Cl1—Sn1—C6	125.60 (5)	C4—C3—C7	122.5 (2)
Cl1^i^—Sn1—C1	78.92 (5)	C3—C4—C5	120.1 (2)
Cl1^i^—Sn1—C2	96.51 (5)	C3—C4—C8	119.6 (2)
Cl1^i^—Sn1—C3	122.99 (5)	C5—C4—C8	120.4 (2)
Cl1^i^—Sn1—C4	131.87 (5)	C6—C5—C4	120.4 (2)
Cl1^i^—Sn1—C5	113.02 (5)	C6—C5—C9	119.9 (2)
Cl1^i^—Sn1—C6	87.77 (5)	C4—C5—C9	119.7 (2)
Cl2—Sn1—C1	160.46 (5)	C1—C6—C5	118.6 (2)
Cl2—Sn1—C2	168.90 (5)	C1—C6—C10	118.9 (3)
Cl2—Sn1—C3	151.42 (5)	C5—C6—C10	122.5 (3)
Cl2—Sn1—C4	138.73 (5)	C3—C7—H71	109.5
Cl2—Sn1—C5	135.45 (5)	C3—C7—H72	109.5
Cl2—Sn1—C6	143.03 (5)	H71—C7—H72	109.5
Cl3—Sn1—C1	99.07 (6)	C3—C7—H73	109.5
Cl3—Sn1—C2	124.60 (5)	H71—C7—H73	109.5
Cl3—Sn1—C3	124.44 (4)	H72—C7—H73	109.5
Cl3—Sn1—C4	100.80 (5)	C4—C8—H81	109.5
Cl3—Sn1—C5	78.78 (5)	C4—C8—H82	109.5
Cl3—Sn1—C6	76.71 (5)	H81—C8—H82	109.5
Cl4^ii^—Sn1—C1	121.84 (5)	C4—C8—H83	109.5
Cl4^ii^—Sn1—C2	96.29 (5)	H81—C8—H83	109.5
Cl4^ii^—Sn1—C3	74.39 (5)	H82—C8—H83	109.5
Cl4^ii^—Sn1—C4	76.28 (5)	C5—C9—H91	109.5
Cl4^ii^—Sn1—C5	98.30 (5)	C5—C9—H92	109.5
Cl4^ii^—Sn1—C6	122.18 (5)	H91—C9—H92	109.5
Sn1—Cl1—Sn1^i^	100.247 (18)	C5—C9—H93	109.5
Sn1—C1—H1	110.9	H91—C9—H93	109.5
Sn1—C2—H2	111.8	H92—C9—H93	109.5
Sn1—C3—C7	119.14 (16)	C6—C10—H101	109.5
Sn1—C4—C8	123.29 (18)	C6—C10—H102	109.5
Sn1—C5—C9	122.01 (19)	H101—C10—H102	109.5
Sn1—C6—C10	116.23 (17)	C6—C10—H103	109.5
Al1—Cl2—Sn1	95.56 (3)	H101—C10—H103	109.5
Al1—Cl3—Sn1	90.68 (3)	H102—C10—H103	109.5

Symmetry codes: (i) −*x*+1, −*y*, −*z*+1; (ii) −*x*+1, −*y*+1, −*z*+1.

### catena-Poly[[chloridoaluminate(III)]-tri-µ-chlorido-4':1κ2Cl,1:2κ4Cl-[(η6-1,2,3,4-tetramethylbenzene)tin(II)]-di-µ-chlorido-2:3κ4Cl-[(η6-1,2,3,4-tetramethylbenzene)tin(II)]-di-µ-chlorido-3:4κ4Cl-[chloridoaluminate(III)]-µ-chlorido-4:1'κ2Cl] (I) Hydrogen-bond geometry (Å, º)

**Table:**

*D*—H···*A*	*D*—H	H···*A*	*D*···*A*	*D*—H···*A*
C10—H103···Cl3	0.97	2.79	3.633 (3)	146
C7—H71···Cl4^ii^	0.97	2.80	3.622 (3)	144

Symmetry code: (ii) −*x*+1, −*y*+1, −*z*+1.

### catena-Poly[[chloridogallate(III)]-tri-µ-chlorido-4':1κ2Cl,1:2κ4Cl-[(η6-1,2,3,4-tetramethylbenzene)tin(II)]-di-µ-chlorido-2:3κ4Cl-[(η6-1,2,3,4-tetramethylbenzene)tin(II)]-di-µ-chlorido-3:4κ4Cl-[chloridogallate(III)]-µ-chlorido-4:1'κ2Cl] (II) Crystal data

**Table:**

[Ga_2_Sn_2_Cl_10_(C_10_H_14_)_2_]	*Z* = 1
*M**_r_* = 999.78	*F*(000) = 480
Triclinic, *P*1	*D*_x_ = 1.931 Mg m^−^^3^
*a* = 8.7572 (4) Å	Mo *K*α radiation, λ = 0.71073 Å
*b* = 9.1310 (4) Å	Cell parameters from 21911 reflections
*c* = 11.2966 (5) Å	θ = 4.5–59.3°
α = 85.424 (3)°	µ = 3.77 mm^−^^1^
β = 72.805 (3)°	*T* = 213 K
γ = 86.886 (4)°	Needle, colourless
*V* = 859.73 (7) Å^3^	0.61 × 0.13 × 0.03 mm

### catena-Poly[[chloridogallate(III)]-tri-µ-chlorido-4':1κ2Cl,1:2κ4Cl-[(η6-1,2,3,4-tetramethylbenzene)tin(II)]-di-µ-chlorido-2:3κ4Cl-[(η6-1,2,3,4-tetramethylbenzene)tin(II)]-di-µ-chlorido-3:4κ4Cl-[chloridogallate(III)]-µ-chlorido-4:1'κ2Cl] (II) Data collection

**Table:**

Stoe IPDS 2 diffractometer	4242 reflections with *I* > 2σ(*I*)
ω scans	*R*_int_ = 0.048
Absorption correction: multi-scan (Blessing, 1995)	θ_max_ = 29.2°, θ_min_ = 2.2°
*T*_min_ = 0.376, *T*_max_ = 0.656	*h* = −11→11
17522 measured reflections	*k* = −12→11
4638 independent reflections	*l* = −15→15

### catena-Poly[[chloridogallate(III)]-tri-µ-chlorido-4':1κ2Cl,1:2κ4Cl-[(η6-1,2,3,4-tetramethylbenzene)tin(II)]-di-µ-chlorido-2:3κ4Cl-[(η6-1,2,3,4-tetramethylbenzene)tin(II)]-di-µ-chlorido-3:4κ4Cl-[chloridogallate(III)]-µ-chlorido-4:1'κ2Cl] (II) Refinement

**Table:**

Refinement on *F*^2^	Primary atom site location: structure-invariant direct methods
Least-squares matrix: full	Hydrogen site location: inferred from neighbouring sites
*R*[*F*^2^ > 2σ(*F*^2^)] = 0.033	H-atom parameters constrained
*wR*(*F*^2^) = 0.069	*w* = 1/[σ^2^(*F*_o_^2^) + (0.0176*P*)^2^ + 0.9105*P*] where *P* = (*F*_o_^2^ + 2*F*_c_^2^)/3
*S* = 1.22	(Δ/σ)_max_ = 0.001
4638 reflections	Δρ_max_ = 0.71 e Å^−^^3^
158 parameters	Δρ_min_ = −0.50 e Å^−^^3^
0 restraints	

### catena-Poly[[chloridogallate(III)]-tri-µ-chlorido-4':1κ2Cl,1:2κ4Cl-[(η6-1,2,3,4-tetramethylbenzene)tin(II)]-di-µ-chlorido-2:3κ4Cl-[(η6-1,2,3,4-tetramethylbenzene)tin(II)]-di-µ-chlorido-3:4κ4Cl-[chloridogallate(III)]-µ-chlorido-4:1'κ2Cl] (II) Special details

**Table:**

Geometry. All esds (except the esd in the dihedral angle between two l.s. planes) are estimated using the full covariance matrix. The cell esds are taken into account individually in the estimation of esds in distances, angles and torsion angles; correlations between esds in cell parameters are only used when they are defined by crystal symmetry. An approximate (isotropic) treatment of cell esds is used for estimating esds involving l.s. planes.

### catena-Poly[[chloridogallate(III)]-tri-µ-chlorido-4':1κ2Cl,1:2κ4Cl-[(η6-1,2,3,4-tetramethylbenzene)tin(II)]-di-µ-chlorido-2:3κ4Cl-[(η6-1,2,3,4-tetramethylbenzene)tin(II)]-di-µ-chlorido-3:4κ4Cl-[chloridogallate(III)]-µ-chlorido-4:1'κ2Cl] (II) Fractional atomic coordinates and isotropic or equivalent isotropic displacement parameters (Å^2^)

**Table:**

	*x*	*y*	*z*	*U*_iso_*/*U*_eq_	
Sn1	0.52200 (3)	0.16597 (2)	0.60194 (2)	0.02838 (6)	
Ga1	0.78103 (4)	0.41311 (3)	0.32615 (3)	0.03202 (8)	
Cl1	0.29875 (9)	0.01494 (8)	0.55911 (7)	0.03389 (15)	
Cl2	0.52418 (10)	0.36331 (9)	0.37434 (8)	0.03987 (17)	
Cl3	0.86231 (14)	0.32600 (11)	0.48157 (11)	0.0565 (3)	
Cl4	0.80145 (11)	0.65004 (8)	0.30948 (10)	0.0477 (2)	
Cl5	0.90567 (15)	0.31991 (13)	0.15424 (11)	0.0646 (3)	
C1	0.6226 (4)	−0.0506 (4)	0.7670 (3)	0.0372 (7)	
H1	0.670505	−0.133397	0.724495	0.045*	
C2	0.4589 (4)	−0.0435 (3)	0.8188 (3)	0.0332 (6)	
H2	0.397444	−0.121308	0.810744	0.040*	
C3	0.3829 (4)	0.0773 (3)	0.8831 (3)	0.0301 (6)	
C4	0.4772 (4)	0.1935 (4)	0.8922 (3)	0.0331 (6)	
C5	0.6443 (4)	0.1853 (4)	0.8391 (3)	0.0345 (6)	
C6	0.7182 (4)	0.0618 (4)	0.7763 (3)	0.0358 (7)	
C7	0.2046 (4)	0.0785 (4)	0.9400 (3)	0.0431 (8)	
H71	0.156274	0.160251	0.902162	0.065*	
H72	0.179189	0.089092	1.028578	0.065*	
H73	0.163275	−0.012949	0.926109	0.065*	
C8	0.3983 (5)	0.3255 (4)	0.9602 (4)	0.0503 (9)	
H81	0.426066	0.413765	0.905725	0.075*	
H82	0.434745	0.332000	1.032732	0.075*	
H83	0.283145	0.315915	0.986078	0.075*	
C9	0.7449 (5)	0.3105 (5)	0.8505 (4)	0.0570 (11)	
H91	0.857192	0.285182	0.814368	0.085*	
H92	0.725155	0.327596	0.937538	0.085*	
H93	0.716585	0.398926	0.807067	0.085*	
C10	0.8972 (4)	0.0473 (5)	0.7176 (4)	0.0492 (9)	
H101	0.923751	−0.046929	0.681388	0.074*	
H102	0.951087	0.053946	0.780598	0.074*	
H103	0.931353	0.125718	0.653377	0.074*	

### catena-Poly[[chloridogallate(III)]-tri-µ-chlorido-4':1κ2Cl,1:2κ4Cl-[(η6-1,2,3,4-tetramethylbenzene)tin(II)]-di-µ-chlorido-2:3κ4Cl-[(η6-1,2,3,4-tetramethylbenzene)tin(II)]-di-µ-chlorido-3:4κ4Cl-[chloridogallate(III)]-µ-chlorido-4:1'κ2Cl] (II) Atomic displacement parameters (Å^2^)

**Table:**

	*U*^11^	*U*^22^	*U*^33^	*U*^12^	*U*^13^	*U*^23^
Sn1	0.03005 (11)	0.02753 (10)	0.02689 (10)	−0.00228 (7)	−0.00647 (8)	−0.00417 (7)
Ga1	0.03199 (18)	0.02486 (15)	0.03540 (18)	−0.00005 (13)	−0.00417 (14)	−0.00194 (13)
Cl1	0.0231 (3)	0.0373 (4)	0.0388 (4)	−0.0013 (3)	−0.0023 (3)	−0.0140 (3)
Cl2	0.0340 (4)	0.0420 (4)	0.0427 (4)	−0.0057 (3)	−0.0091 (3)	−0.0031 (3)
Cl3	0.0597 (6)	0.0533 (5)	0.0652 (6)	−0.0163 (5)	−0.0344 (5)	0.0165 (5)
Cl4	0.0363 (4)	0.0250 (3)	0.0707 (6)	−0.0008 (3)	−0.0001 (4)	0.0021 (3)
Cl5	0.0596 (6)	0.0660 (6)	0.0553 (6)	0.0052 (5)	0.0073 (5)	−0.0248 (5)
C1	0.0423 (18)	0.0354 (16)	0.0311 (15)	0.0067 (14)	−0.0080 (14)	−0.0029 (12)
C2	0.0396 (17)	0.0321 (14)	0.0291 (14)	−0.0074 (12)	−0.0116 (13)	0.0016 (11)
C3	0.0278 (14)	0.0381 (15)	0.0240 (13)	−0.0017 (12)	−0.0076 (11)	0.0011 (11)
C4	0.0346 (16)	0.0394 (15)	0.0254 (13)	0.0001 (12)	−0.0081 (12)	−0.0072 (11)
C5	0.0317 (15)	0.0440 (17)	0.0309 (14)	−0.0068 (13)	−0.0128 (13)	−0.0033 (12)
C6	0.0279 (15)	0.0471 (17)	0.0307 (14)	0.0038 (13)	−0.0076 (12)	0.0016 (13)
C7	0.0293 (16)	0.058 (2)	0.0371 (17)	−0.0052 (15)	−0.0031 (14)	0.0044 (15)
C8	0.052 (2)	0.048 (2)	0.050 (2)	0.0029 (17)	−0.0109 (18)	−0.0217 (17)
C9	0.047 (2)	0.065 (3)	0.064 (3)	−0.0206 (19)	−0.018 (2)	−0.013 (2)
C10	0.0277 (17)	0.066 (2)	0.049 (2)	0.0050 (16)	−0.0084 (15)	0.0106 (18)

### catena-Poly[[chloridogallate(III)]-tri-µ-chlorido-4':1κ2Cl,1:2κ4Cl-[(η6-1,2,3,4-tetramethylbenzene)tin(II)]-di-µ-chlorido-2:3κ4Cl-[(η6-1,2,3,4-tetramethylbenzene)tin(II)]-di-µ-chlorido-3:4κ4Cl-[chloridogallate(III)]-µ-chlorido-4:1'κ2Cl] (II) Geometric parameters (Å, º)

**Table:**

Sn1—Cl1	2.6299 (8)	C2—H2	0.9400
Sn1—Cl1^i^	2.6481 (7)	C3—C4	1.408 (4)
Sn1—Cl2	3.0155 (9)	C3—C7	1.503 (4)
Sn1—Cl3	3.2597 (11)	C4—C5	1.408 (5)
Sn1—Cl4^ii^	3.1499 (9)	C4—C8	1.505 (5)
Sn1—C1	2.891 (3)	C5—C6	1.404 (5)
Sn1—C2	2.921 (3)	C5—C9	1.517 (5)
Sn1—C3	3.104 (3)	C6—C10	1.513 (5)
Sn1—C4	3.214 (3)	C7—H71	0.9700
Sn1—C5	3.185 (3)	C7—H72	0.9700
Sn1—C6	3.043 (3)	C7—H73	0.9700
Sn1—Ga1	3.8987 (4)	C8—H81	0.9700
Sn1—Sn1^i^	4.0503 (4)	C8—H82	0.9700
Ga1—Cl2	2.2159 (9)	C8—H83	0.9700
Ga1—Cl3	2.1625 (10)	C9—H91	0.9700
Ga1—Cl4	2.1691 (8)	C9—H92	0.9700
Ga1—Cl5	2.1439 (10)	C9—H93	0.9700
C1—C2	1.379 (5)	C10—H101	0.9700
C1—C6	1.388 (5)	C10—H102	0.9700
C1—H1	0.9400	C10—H103	0.9700
C2—C3	1.395 (4)		
			
Cl1—Sn1—Cl1^i^	79.76 (2)	Cl5—Ga1—Cl2	109.01 (4)
Cl1—Sn1—Cl2	88.37 (3)	Cl3—Ga1—Cl2	106.43 (4)
Cl1^i^—Sn1—Cl2	84.56 (3)	Cl4—Ga1—Cl2	108.21 (4)
Cl1—Sn1—Cl4^ii^	73.00 (2)	Sn1—Cl1—Sn1^i^	100.24 (2)
Cl1^i^—Sn1—Cl4^ii^	147.47 (3)	Ga1—Cl2—Sn1	95.14 (3)
Cl1—Sn1—Cl3	146.02 (3)	Ga1—Cl3—Sn1	89.58 (3)
Cl1^i^—Sn1—Cl3	74.35 (3)	Ga1—Cl4—Sn1^ii^	115.87 (3)
Cl2—Sn1—Cl3	67.82 (2)	C2—C1—C6	121.5 (3)
Cl2—Sn1—Cl4^ii^	77.33 (2)	C2—C1—H1	119.2
Cl4^ii^—Sn1—Cl3	121.37 (3)	C6—C1—H1	119.2
Cl1—Sn1—C1	98.21 (7)	C1—C2—C3	121.1 (3)
Cl1^i^—Sn1—C1	79.15 (7)	C1—C2—H2	119.5
Cl1—Sn1—C2	80.68 (7)	C3—C2—H2	119.5
Cl1^i^—Sn1—C2	96.61 (7)	C2—C3—C4	118.4 (3)
Cl1—Sn1—C3	88.88 (6)	C2—C3—C7	119.1 (3)
Cl1^i^—Sn1—C3	123.05 (6)	C4—C3—C7	122.5 (3)
Cl1—Sn1—C4	113.52 (6)	C3—C4—C5	120.1 (3)
Cl1^i^—Sn1—C4	131.84 (6)	C3—C4—C8	119.5 (3)
Cl1—Sn1—C5	133.20 (6)	C5—C4—C8	120.4 (3)
Cl1^i^—Sn1—C5	112.86 (6)	C6—C5—C4	120.4 (3)
Cl1—Sn1—C6	125.01 (7)	C6—C5—C9	119.9 (3)
Cl1^i^—Sn1—C6	87.73 (7)	C4—C5—C9	119.7 (3)
Cl2—Sn1—C1	161.00 (7)	C1—C6—C5	118.4 (3)
Cl2—Sn1—C2	168.57 (7)	C1—C6—C10	119.0 (3)
Cl2—Sn1—C3	151.20 (6)	C5—C6—C10	122.6 (3)
Cl2—Sn1—C4	138.75 (6)	C3—C7—H71	109.5
Cl2—Sn1—C5	135.87 (6)	C3—C7—H72	109.5
Cl2—Sn1—C6	143.69 (7)	H71—C7—H72	109.5
C1—Sn1—Cl3	98.11 (7)	C3—C7—H73	109.5
C2—Sn1—Cl3	123.48 (7)	H71—C7—H73	109.5
C3—Sn1—Cl3	123.69 (6)	H72—C7—H73	109.5
C4—Sn1—Cl3	100.17 (6)	C4—C8—H81	109.5
C5—Sn1—Cl3	78.09 (6)	C4—C8—H82	109.5
C6—Sn1—Cl3	75.93 (7)	H81—C8—H82	109.5
C1—Sn1—Cl4^ii^	121.62 (7)	C4—C8—H83	109.5
C2—Sn1—Cl4^ii^	96.19 (7)	H81—C8—H83	109.5
C3—Sn1—Cl4^ii^	74.45 (6)	H82—C8—H83	109.5
Cl4^ii^—Sn1—C4	76.64 (6)	C5—C9—H91	109.5
Cl4^ii^—Sn1—C5	98.82 (7)	C5—C9—H92	109.5
C6—Sn1—Cl4^ii^	122.46 (6)	H91—C9—H92	109.5
Sn1—C1—H1	110.6	C5—C9—H93	109.5
Sn1—C2—H2	111.6	H91—C9—H93	109.5
Sn1—C3—C7	119.1 (2)	H92—C9—H93	109.5
Sn1—C4—C8	123.0 (2)	C6—C10—H101	109.5
Sn1—C5—C9	121.9 (2)	C6—C10—H102	109.5
Sn1—C6—C10	116.1 (2)	H101—C10—H102	109.5
Cl5—Ga1—Cl3	113.84 (5)	C6—C10—H103	109.5
Cl5—Ga1—Cl4	110.73 (5)	H101—C10—H103	109.5
Cl3—Ga1—Cl4	108.39 (4)	H102—C10—H103	109.5

Symmetry codes: (i) −*x*+1, −*y*, −*z*+1; (ii) −*x*+1, −*y*+1, −*z*+1.

### catena-Poly[[chloridogallate(III)]-tri-µ-chlorido-4':1κ2Cl,1:2κ4Cl-[(η6-1,2,3,4-tetramethylbenzene)tin(II)]-di-µ-chlorido-2:3κ4Cl-[(η6-1,2,3,4-tetramethylbenzene)tin(II)]-di-µ-chlorido-3:4κ4Cl-[chloridogallate(III)]-µ-chlorido-4:1'κ2Cl] (II) Hydrogen-bond geometry (Å, º)

**Table:**

*D*—H···*A*	*D*—H	H···*A*	*D*···*A*	*D*—H···*A*
C10—H103···Cl3	0.97	2.74	3.605 (4)	149
C7—H71···Cl4^ii^	0.97	2.78	3.612 (4)	144

Symmetry code: (ii) −*x*+1, −*y*+1, −*z*+1.
